# MSI2 deficiency in ILC3s attenuates DSS-induced colitis by affecting the intestinal microbiota

**DOI:** 10.3389/fimmu.2022.963379

**Published:** 2023-01-12

**Authors:** Nengneng Li, Shiquan Xu, Shuaishuai Zhang, Qiang Zhu, Xiaole Meng, Wenbin An, Baoqing Fu, Mengya Zhong, Yan Yang, Zeyang Lin, Xueni Liu, Junjie Xia, Jie Wang, Tingting You, Changxiu Yan, Huamei Tang, Guohong Zhuang, Zhihai Peng

**Affiliations:** ^1^ Department of Organ Transplantation, Xiang'an Hospital of Xiamen University, School of Medicine, Xiamen University, Xiamen, China; ^2^ Fujian Provincial Key Laboratory of Organ and Tissue Regeneration, Organ Transplantation Institute of Xiamen University, School of Medicine, Xiamen University, Xiamen, Fujian, China; ^3^ Department of Laboratory Medicine, Xiang’an Hospital, Xiamen University, Xiamen, Fujian, China; ^4^ Cancer Research Center, School of Medicine, Xiamen University, Xiamen, Fujian, China; ^5^ Department of Pathology, Zhongshan Hospital, Xiamen University, Xiamen, Fujian, China; ^6^ Department of General Surgery, Shanghai General Hospital, School of Medicine, Shanghai Jiao Tong University, Shanghai, China; ^7^ Department of Pathology, Xiang’an Hospital, School of Medicine, Xiamen University, Xiamen, Fujian, China; ^8^ Organ Transplantation Clinical Medical Center of Xiamen University, Xiamen, Fujian, China

**Keywords:** inflammatory bowel disease, intestinal microbiota, Musashi2, group 3 innate lymphoid cells, Lactobacillaceae

## Abstract

**Background:**

The etiology and pathogenesis of inflammatory bowel disease (IBD), including ulcerative colitis (UC) and Crohn’s disease (CD), are generally believed to be related to immune dysfunction and intestinal microbiota disorder. However, the exact mechanism is not yet fully understood. The pathological changes associated with dextran sodium sulfate (DSS)-induced colitis are similar to those in human UC. As a subgroup of the innate immune system, group 3 innate lymphoid cells (ILC3s) are widely distributed in the lamina propria of the intestinal mucosa, and their function can be regulated by a variety of molecules. Musashi2 (MSI2) is a type of evolutionarily conserved RNA-binding protein that maintains the function of various tissue stem cells and is essential for postintestinal epithelial regeneration. The effect of MSI2 deficiency in ILC3s on IBD has not been reported. Thus, mice with conditional MSI2 knockout in ILC3s were used to construct a DSS-induced colitis model and explore its effects on the pathogenesis of IBD and the species, quantity and function of the intestinal microbiota.

**Methods:**

*Msi2^flox/flox^* mice (*Msi2^fl/fl^*) and *Msi2^flox/flox^Rorc^Cre^* mice (*Msi2^ΔRorc^*) were induced by DSS to establish the IBD model. The severity of colitis was evaluated by five measurements: body weight percentage, disease activity index, colon shortening degree, histopathological score and routine blood examination. The species, quantity and function of the intestinal microbiota were characterized by high-throughput 16S rRNA gene sequencing of DNA extracted from fecal samples.

**Results:**

MSI2 was knocked out in the ILC3s of *Msi2^ΔRorc^* mice. The *Msi2^ΔRorc^* mice exhibited reductions in body weight loss, the disease activity index, degree of colon shortening, tissue histopathological score and immune cells in the peripheral blood compared to those of *Msi2^fl/fl^* mice after DSS administration. The 16S rRNA sequencing results showed that the diversity of the intestinal microbiota in DSS-treated *Msi2^ΔRorc^* mice changed, with the abundance of Firmicutes increasing and that of Bacteroidetes decreasing. The linear discriminant analysis effect size (LEfSe) approach revealed that Lactobacillaceae could be the key bacteria in the *Msi2^ΔRorc^* mouse during the improvement of colitis. Using PICRUST2 to predict the function of the intestinal microbiota, it was found that the functions of differential bacteria inferred by modeling were mainly enriched in infectious diseases, immune system and metabolic functions.

**Conclusions:**

MSI2 deficiency in ILC3s attenuated DSS-induced colonic inflammation in mice and affected intestinal microbiota diversity, composition, and function, with Lactobacillaceae belonging to the phylum Firmicutes possibly representing the key bacteria. This finding could contribute to our understanding of the pathogenesis of IBD and provide new insights for its clinical diagnosis and treatment.

## Graphical Abstract

A role for the RNA-binding protein MSI2 in ILC3s, which are master regulators that maintain the balance between the intestinal microbiota and immunity in the colon, has been revealed.

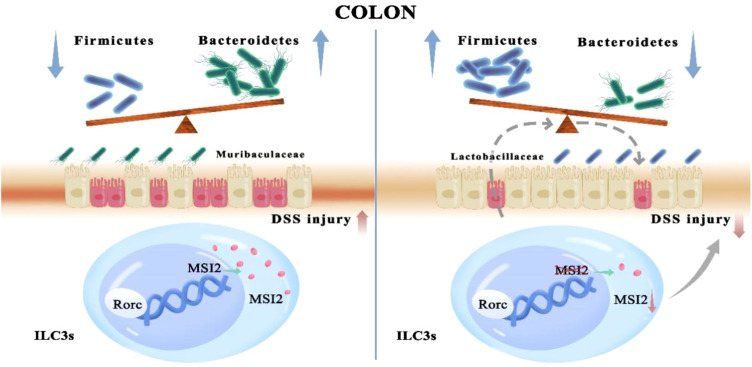

## Introduction

Inflammatory bowel disease (IBD), including ulcerative colitis (UC) and Crohn’s disease (CD), is a chronic inflammatory intestinal disease caused by the interaction of multiple factors, such as heredity, the environment, immunity, the intestinal microbiota, and mental health (1). The role of the intestinal microbiota in the occurrence and development of IBD should not be ignored. There is a very complex relationship between intestinal microbiota imbalance and IBD. Indeed, intestinal microbiota imbalance could be a predisposing factor for IBD, and IBD is also believed to exacerbate intestinal microbiota imbalance, but the interaction mechanism is still unclear (2). In the presence of commensal flora and myeloid cells, DSS can increase the permeability of the intestinal and destroy the surface barrier of the intestinal mucosa of mice, which has become an important means for studying the etiology and pathogenesis of UC, as the DSS-induced colitis model is both stable and reliable (3).

Innate lymphoid cells (ILCs) are a type of innate immune cell discovered in recent years that are derived from common lymphoid progenitor cells (CLPs). They belong to the lymphocyte lineage and have a typical lymphocyte morphology. In addition, they lack antigen-specific recognition receptors on the surface and play an important role in the inflammatory response, immune defense and tissue repair (4). According to the transcription factors expressed and the cytokines secreted by these cells, ILCs are divided into four subgroups, T-bet^+^ group 1 ILCs (ILC1s), GATA3^+^ group 2 ILCs (ILC2s), retinoid-related orphan receptor (RORγt^+^) group 3 ILCs (ILC3s) and regulatory innate lymphoid cells (ILCregs), which are “mirror cells” associated with Th1, Th2, Th17 and Treg cells (5). Among them, ILC3s are abundantly distributed in the intestinal mucosa and maintain homeostasis by coordinating lymphoid tissue development, commensal bacterial regulation, tissue repair, host defense, and adaptive immune regulation (6). Under physiological conditions, ILC3s can maintain the homeostasis of the intestinal mucosal microenvironment by secreting appropriate amounts of IL-22, IL-17, and GM-CSF to protect intestinal epithelial cells from microbial invasion. However, under pathological conditions, the abnormal quantity or function of ILC3s and their transformation into ILC1s can cause excessive secretion of IL-22, IL-17 and IFN-γ, thereby promoting the aggravation and malignant transformation of IBD (7, 8).

The Musashi (MSI) family is a class of evolutionarily conserved RNA-binding proteins, including Musashi1 (MSI1) and Musashi2 (MSI2), and is closely related to the regulation of stem and progenitor cells (9). As a key regulatory molecule of hematopoietic stem cells, MSI2 is involved in regulating the asymmetric division, differentiation and self-renewal of stem cells (10). Knockout of MSI2 in embryonic stem cells inhibits their self-renewal capacity (11). Functional research on the MSI family in the gut has just begun, and some studies have reported that knockdown of both types of MSI genes in the intestinal epithelium has no effect on intestinal homeostasis (12). However, upon exposure to high doses of ionizing radiation, MSI expression and activity in the intestinal epithelium is necessary for epithelial regeneration after injury (13). In addition, MSI2 is overexpressed in a variety of solid tumors and promotes tumor progression, such as that in colon cancer, lung cancer, pancreatic cancer, and liver cancer (14). However, whether MSI2 plays a role in IBD or has regulatory effects on innate and adaptive immunity has not been reported, and whether MSI2 deletion in ILC3s can alter the immune response and affect the composition of the intestinal microbiota remains unknown.

In this study, a DSS-induced colitis model was constructed in mice with conditional knockout of MSI2 in ILC3s to explore its effects on the pathogenesis of IBD and the type, quantity and function of the intestinal microbiota, among which Lactobacillaceae among the Firmicutes could be the key bacterial taxon. This study will provide new clues for the pathogenesis, clinical diagnosis and treatment of IBD.

## Materials and methods

### Animals

Induced conditional knockout of the MSI2 gene in RORγt^+^ ILC3s and T cells in C57BL/6J mice (*Msi2 ^f1ox/f1ox^Rorc ^Cre^*) was established using the Cre/LoxP system. All mice were housed under specific pathogen-free conditions in the Xiamen University Laboratory Animal Center.

The mice were given a standard diet and drinking water and were used for follow-up experiments at 8 weeks of age. Animal experiments followed the ARRIVE guidelines and were approved by the Animal Ethics Committee of the Xiamen University Laboratory Animal Center.

### Establishment and evaluation of DSS-induced colitis

Eight-week-old male mice with similar body weights were selected for the experiment, and the mice receiving DSS solution in the drinking water were set as the experimental group, including *Msi2^fl/fl^* mice (*Msi2^fl/fl^* DSS) and *Msi2^ΔRorc^* mice (*Msi2^ΔRorc^* DSS). Mice drinking regular water ad libitum were set as the control group, including *Msi2^fl/fl^* mice (*Msi2^fl/fl^* Control) and *Msi2^ΔRorc^* mice (*Msi2^ΔRorc^* Control). The mice in the experimental group were allowed to continuously drink 4% DSS (molecular weight 36-50 kD, MP Biomedicals, Solon, OH) solution for 5 days and then were changed to regular water ad libitum for 2 days to establish the acute colitis model, and the mice in the control group drank regular water ad libitum continuously for 7 days (15). The health status of the mice was observed every day, and the disease activity index (DAI) was scored according to the degree of weight loss, stool characteristics and degree of stool bleeding. The DAI was scored using a unified scoring standard. For weight loss, 0, 1~5, 5~10, 10~15, and >15 was scored as 0, 1, 2, 3, and 4 points, respectively. For stool traits, normal, loose, and watery stool was scored as 0, 2, and 4 points, respectively. For bloody stools, normal, occult blood-positive, and macroscopic blood stools were scored as 0, 2, and 4 points, respectively (16). After modeling, the mice were euthanized, the entire colon and rectum (from the beginning of the cecum to the end of the rectum) were taken and photographed, and the length of the whole large intestine was recorded.

### Flow cytometry

The peripheral lymph nodes (brachial plexus, axillary, and inguinal lymph nodes), mesenteric lymph nodes and spleen were removed from the dissected mice, placed in staining buffer (2% fetal bovine serum and 0.2% penicillin/streptomycin in 1× PBS solution) and ground through a strainer to collect the single-cell suspension in a centrifuge tube with the lysing of red blood cells in spleen cells. The colon was collected, mesenteric adipose tissue and Pyle lymph nodes were removed, and then the contents were cut longitudinally and rinsed away. Two extraction washes were performed with PBS containing 1 mM DTT (Roche) and 5 mM EDTA (Solarbio) for a total of 20 min to remove intraepithelial lymphocytes. Laminar propria immune cells were isolated with RPMI 1640 containing 200 U/ml collagenase VIII (Sigma Aldrich) and 0.15 mg/ml DNase I (Roche) for 90 min at 37°C with shaking, and lymphocytes were enriched at the interface between a gradient of 40% and 80% Percoll (Cytiva). The cells were washed and counted under a microscope. Single-cell suspensions were stained with a combination of fluorescently labeled monoclonal antibodies. For cell surface molecules, the following antibodies were used for staining: Fixable Viability-eFluor780, Lineage markers-fluorescein isothiocyanate (FITC), CD45-PerCP, and CD127-phycoerythrin (PE)-Cyanine7. For intracellular molecular staining, cells were fixed and permeabilized and then stained with the following antibodies: RORγt-Brilliant Violet (BV) 421, MSI2 rabbit anti-mouse antibody and Alexa Fluor 647 goat anti-rabbit antibody. Data were acquired with a Cytoflex LX (Beckman Coulter) multicolor flow cytometer and analyzed with FlowJo_V10 software (Tree Star) (17).

### HE staining

After the modeling, a part of the terminal colon of the mouse was taken and washed with precooled PBS solution. Then, the sample was fixed in 4% paraformaldehyde and embedded in paraffin. After dehydration and clearing, the tissues were immersed in wax and then cut into 5-µm-thick sections. The sections were then dewaxed and stained with hematoxylin and eosin for histological studies according to standard histological procedures. The cells were observed and photographed under a microscope after mounting the coverslips. Histopathological scoring was performed according to the degree of epithelial damage and inflammatory cell infiltration (18).

### Blood cell counts

Before euthanizing the mice in the DSS-treated experimental group, they were anesthetized with a gas anesthesia machine equipped with isoflurane. After euthanasia, one of the eyeballs was rapidly removed, and blood was dripped into an anticoagulation tube. An automatic blood cell analyzer (BM830) was used for routine blood testing.

### Genomic DNA extraction and PCR

The toes and tails of the mice were clipped for numbering and genotype identification, respectively. Mouse tail lysate and protease were used to lyse and digest mouse tails to extract total DNA. Then, the target gene fragments were amplified by PCR. The *Msi2^fl/fl^* gene identification primers were as follows: Primer 1, 5’-GTCTGGGCATTGACAGCCCTGTTT-3’; Primer 2, 5’-CTGAGCCCAGAAAGCGAAGGA-3’; and Primer 3, 5’-AACCTTGGCTTGGCAGCGTCTGAA-3’. The *Rorc-Cre* gene identification primers were as follows: Primer 1, 5’-CGATGCAACGAGTGATGAGG-3’, and Primer 2, 5’-GCATTGCTGTCACTTGGTCGT-3’. The molecular weight of the amplified product was analyzed by agarose gel electrophoresis (19).

The genomic DNA of fecal samples was extracted by the CTAB method, and the purity and concentration of the extracted DNA were detected by agarose gel electrophoresis. Then, an appropriate amount of DNA was placed into a centrifuge tube and diluted with sterile water to 1 ng/μl (20). The diluted genomic DNA was used as a template, and specific primers were selected with barcodes, Phusion^®^ High-Fidelity PCR Master Mix with GC Buffer (New England Biolabs) and high-efficiency high-fidelity enzymes (New England Biolabs) to amplify target genes *in vitro* according to the amplification region. The primers used to identify bacterial diversity corresponded to the 16S V4 region (515F and 806R). Agarose gel electrophoresis was used to detect the amplification products, and the PCR products were mixed in equidense ratios. Then, mixed PCR products were purified with a Qiagen Gel Extraction Kit (Qiagen, Germany) (21).

### Library construction and on-board sequencing

The library was constructed using the NEBNext^®^ Ultra™ II DNA Library Prep Kit and then quantified by Qubit and q-PCR. After the library was qualified, NovaSeq6000 was run for on-board detection.

### Bioinformatics analysis

The data of each sample were distinguished from the original data analyzed by the machine based on the barcode sequence and PCR amplification primer sequence. After truncating the barcode and primer sequences, FLASH software was used to splice the reads of the sample to obtain raw tags. Then, the obtained raw tags were analyzed for quality control by Fastp software to obtain high-quality clean tags. Finally, Usearch software was used to compare the clean tags with the database to detect and remove chimeras to obtain the final effective data, that is, the effective tags (22). The DADA2 module of QIIME2 software was used to denoise the effective tags and filter out sequences with an abundance of less than 5 to finally obtain amplicon sequence variants (ASVs) and their feature tables. Subsequently, the ASVs obtained using the classify-sklearn module in QIIME2 software were compared with the database to obtain the species information of each ASV (23).

Alpha diversity analysis was performed, and the rarefaction curve was drawn by using QIIME2 software. The indices for calculating community richness were Observed_otus, Chao1 and dominance; the indices for calculating community diversity were the Shannon and Simpson indices; the index for calculating the sequencing depth was coverage; and the index for calculating species evenness was Pielou_e (24). Subsequently, beta diversity analysis and UniFrac distance calculations were performed using QIIME2 software. Principal component analysis (PCA), principal coordinate analysis (PCoA) and nonmetric multidimensional scaling (NMDS) dimensionality reduction maps were drawn using R software. The maps of the PCA and PCoA results were created with the Ade4 and Ggplot2 packages in R software. The Adonis and ANOSIM functions in QIIME2 software were used to analyze the significance of community structure differences between groups (25). Finally, analysis of significant differences between groups was performed using the linear discriminant analysis effect size (LEfSe) method or R software. Specifically, the LEfSe method was performed by LEfSe software, and the default linear discriminant analysis (LDA) score threshold was set to 4. T test analysis in R software was used to test for differences and obtain p values between the two comparison groups at the six classification levels of phylum, class, order, family, genus, and species. The species with a p value less than 0.05 were selected as significantly different species between groups (26). PICRUSt2 analysis enabled the prediction of bacterial and archaeal metabolic functions by comparing existing 16S rRNA gene sequencing data with a microbial reference genome database of known metabolic functions (27). Heatmaps of the Kyoto Encyclopedia of Genes and Genomes (KEGG) level 1/2/3 functional pathways were drawn in R (28).

### Statistical analysis

Data are presented as the mean ± SEM, and differences between experimental groups were analyzed with GraphPad Prism 8.0 software (La Jolla, CA, USA). Differences between the two groups were analyzed using two-tailed unpaired Student’s t test. Differences among three or more comparison groups were analyzed with one way ANOVA. Bioinformatics analysis was performed with R software. p < 0.05 was considered to indicate a significant difference, expressed as *p < 0.05, **p < 0.01, ***p < 0.001, and ****p < 0.0001.

## Results

### MSI2 deficiency affects ILC3s in *Msi2^ΔRorc^* mice

**Figure 1:**
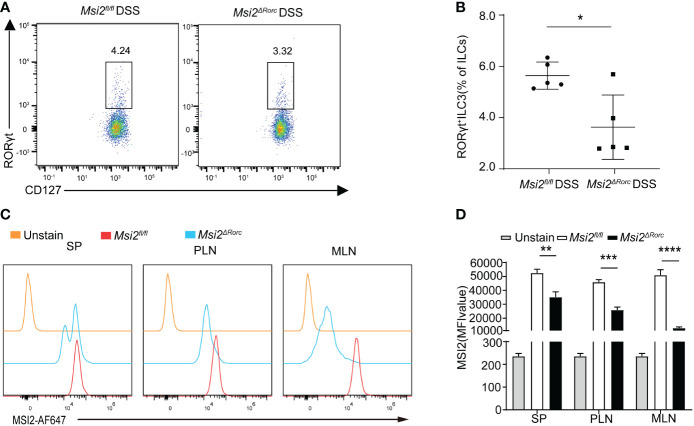
Deletion of MSI2 expression affects the ILC3s of *Msi2^ΔRorc^* mice. **(A, B)** Flow cytometry measured the percentage of ILC3s in the colon of *Msi2^fl/fl^* and *Msi2^ΔRorc^* mice after DSS-induced colitis. **(C, D)** The mean fluorescence intensity of MSI2 in ILC3 cells of the spleen, peripheral lymph nodes and mesenteric lymph nodes in *Msi2^fl/fl^* and *Msi2^ΔRorc^* mice. Data represent the means ± SEMs (n = 5). *p < 0.05, **p < 0.01, ***p < 0.001, ****p < 0.0001.

To investigate the effect of MSI2 deletion in ILC3s on the pathogenesis of IBD, MSI2 was successfully knocked out in ILC3s and first verified. To this end, total DNA was extracted from the tails of *Msi2^fl/fl^* and *Msi2^ΔRorc^* mice, and MSI2 gene expression was verified in *Msi2^ΔRorc^* mice by agarose gel electrophoresis experiments. The experimental results showed that the expression of *Msi2^fl/fl^* could be detected in *Msi2^fl/fl^* and *Msi2^ΔRorc^* mice, and the expression of *Rorc-Cre* could be detected in *Msi2^ΔRorc^* mice, while the expression of *Rorc-Cre* could not be detected in *Msi2^fl/fl^* mice (**Figure S1A**). The *Msi2^fl/fl^* and *Msi2^ΔRorc^* mice were euthanized, and their spleen, peripheral lymph nodes, mesenteric lymph nodes and colon were taken to make single-cell suspensions, which were detected by flow cytometry and then circle gated as shown in **Figure S1B** (29). CD45^+^Lin^-^CD127^+^RORγt^+^ cells represented ILC3 cells, and the mean fluorescence intensity of MSI2 in this population of cells was detected. The results showed that the expression of MSI2 in ILC3s of *Msi2^ΔRorc^* mice was significantly reduced, with the most significant decline in mesenteric lymph nodes, compared with MSI2*^fl/fl^* mice (**Figures 1C, D**). To investigate the effect of MSI2 deletion in ILC3s, we analyzed the quantities of ILC3s in the mouse colon by constructing a DSS-induced colitis model. The data showed that the percentage of ILC3s in *Msi2^ΔRorc^* DSS mice was lower than that in MSI2*^fl/fl^* DSS mice (**Figures 1A, B**). Overall, we concluded that MSI2 deficiency affected the ILC3s of *Msi2^ΔRorc^* mice.

### *Msi2^ΔRorc^* mice are resistant to DSS-induced colitis

**Figure 2:**
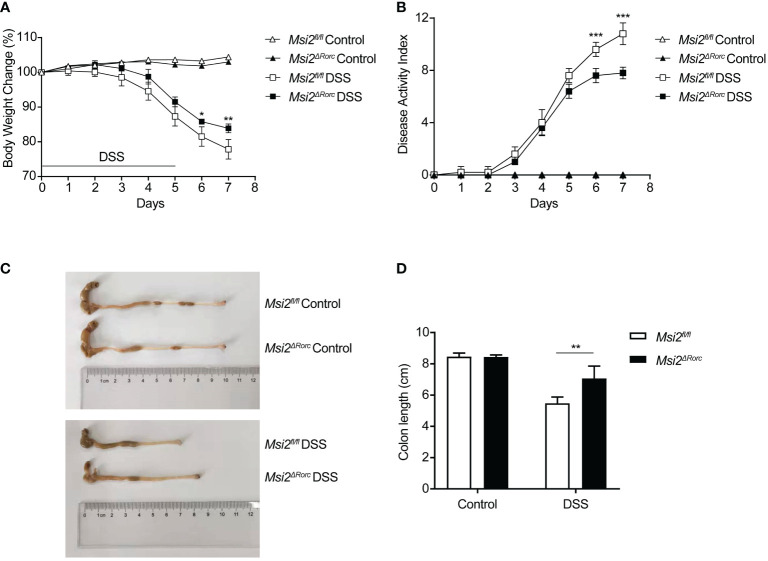
*Msi2^ΔRorc^* mice are resistant to DSS-induced colitis. *Msi2^fl/fl^* and *Msi2^ΔRorc^* mice were fed with 4% DSS or water ad libitum for 5 days followed by recovery on normal drinking water for 2 days prior to euthanasia. **(A)** The body weight change was evaluated daily. **(B)** The disease activity index (DAI) was scored daily. **(C, D)** Representative images of colons from *Msi2^fl/fl^* and *Msi2^ΔRorc^* mice on day 8, when the colon length were examined. Data represent the means ± SEMs (n=5). *p < 0.05, **p < 0.01, ***p < 0.001.

Experimental colitis induced by oral administration of DSS in animals is a well-established model to study the etiology and pathogenesis of UC. To understand the effect of MSI2 deletion in ILC3s on the pathogenesis of UC, mice with MSI2 deletion/normal expression in ILC3s were used to establish a DSS-induced colitis model and explore the function of this gene *in vivo*. Weight loss is a proxy for morbidity. The experimental results showed that the weight loss of mice in the experimental group began to differ on day 6, and the body weight percentages of the *Msi2^fl/fl^* DSS group on days 6 and 7 were 81.5 ± 0.025% and 77.84 ± 0.025%, respectively. The *Msi2^ΔRorc^* DSS group had a reduced degree of body weight loss, with percentages of body weight of 85.8% ± 0.006% and 83.9 ± 0.011% on days 6 and 7, respectively, compared with that of the control (p < 0.05, p < 0.01, **Figure 2A**). The DAI score is an important indicator reflecting the disease activity of colitis. Compared with that of the *Msi2^fl/fl^* DSS group, the DAI score of the *Msi2^ΔRorc^* DSS group began to decrease significantly on day 6 of modeling. DAI scores on days 6 and 7 in the *Msi2^fl/fl^* DSS group were 9.6 ± 0.49 and 10.8 ± 0.748, respectively, while those in the *Msi2^ΔRorc^* DSS group were 7.6 ± 0.49 and 7.8 ± 0.4, respectively (p < 0.001, p < 0.001, **Figure 2B**). There were no significant differences in daily body weight changes or DAI scores among control mice that drank water ad libitum. The colons of mice in the experimental group were significantly shortened. The colon length of the *Msi2^fl/fl^* DSS group was 5.48 ± 0.35 cm, and that of the *Msi2^ΔRorc^* DSS group was 7.06 ± 0.72 cm (p < 0.01, **Figures 2C, D**), showing that the shortening degree of the colon of mice in the *Msi2^ΔRorc^* DSS group was significantly lower than that in the *Msi2^fl/fl^* DSS group and that the difference in colon length between the two groups was 1.58 ± 0.44 cm. There was no significant difference in colon length relative to that of control mice that drank water ad libitum, in which the colon length was 8.45 ± 0.16 cm.

These results suggest that MSI2 deletion in ILC3s can reduce body weight loss, DAI scores, and the degree of colonic shortening after DSS induction.

### Reduced histopathological damage and peripheral leukopenia in *Msi2^ΔRorc^* model mice

DSS induction can increase intestinal permeability, disrupt the intestinal epithelial barrier, activate cytokines and inflammatory pathways and cause intestinal inflammatory responses and damage (30). The intestinal damage resulting from DSS-induced colitis mainly includes two processes: the destruction of epithelial structure and the infiltration of inflammatory cells.

**Figure 3:**
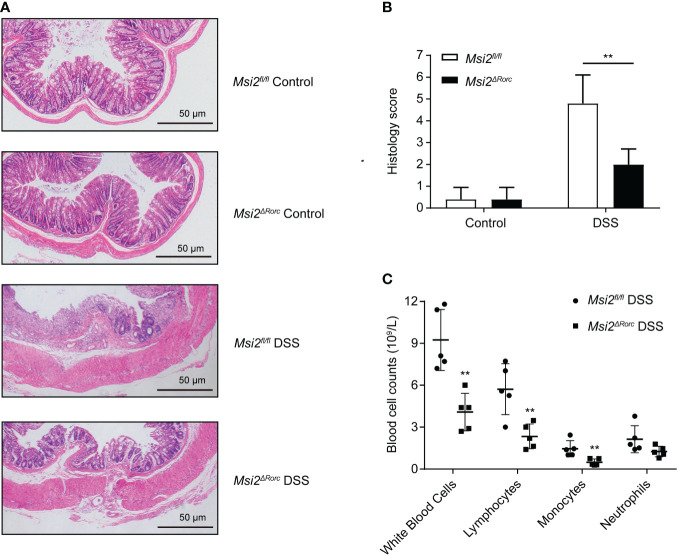
Both histopathological lesions and peripheral blood leukocytes were reduced in the *Msi2^ΔRorc^* mouse model. **(A, B)** *Msi2^fl/fl^* and *Msi2^ΔRorc^* mice were fed 4% DSS or water ad libitum for 5 days, which was followed by recovery on regular drinking water for 2 days prior to euthanasia. Representative images of HE-stained sections of colons (original magnification ×100) from *Msi2^fl/fl^* and *Msi2^ΔRorc^* mice on day 8 and careful examination of the colonic histopathological score. **(C)** The white blood cell counts of both the *Msi2^fl/fl^* DSS group and the *Msi2^ΔRorc^* DSS group were evaluated and identified using an automatic blood cell analyzer BM830 on day 8. Data represent the means ± SEMs (n=5). **p < 0.01.

To study the effect of MSI2 deletion in ILC3s on intestinal injury, the intestinal tissues of mice in each group were fixed as paraffin sections, stained with hematoxylin and eosin, and the histopathologic scores were determined with a unified scoring standard to evaluate the degree of intestinal injury and inflammation in mice. For epithelial injury, normal structures, isolated local lesions, mucosal erosions and ulcers, and extensive lesions deep into the muscularis were scored as 0, 1, 2, and 3 points, respectively. For inflammatory cell infiltration, scores of 0, 1, 2, and 3 points were assigned to no inflammatory cell infiltration, increased inflammatory cells, infiltration of inflammatory cells to the submucosa, and full-thickness infiltration of inflammatory cells in the intestinal wall, respectively (31). In addition, routine blood examination is also a relatively common method at present. Through a blood cell analyzer, the number, proportion and shape of blood cells in peripheral blood were detected to effectively determine whether there was inflammation in the body. To study the effect of MSI2 deletion in ILC3s on the type and number of immune cells, a blood cell analyzer was used to detect the number of immune cells in the peripheral blood of mice. The experimental results showed that the histopathological score of the *Msi2^fl/fl^* DSS group was 4.8 ± 1.17. The intestinal damage was attenuated, and the histopathological score was 2 ± 0.63 in the *Msi2^ΔRorc^* DSS group compared with the control. The difference between the two groups was statistically significant (p < 0.01; **Figures 3A, B**). There was no significant difference in the histopathological scores between the two groups of mice drinking water ad libitum. Routine blood examination showed that the numbers of leukocytes, lymphocytes and monocytes in the peripheral blood of the *Msi2^fl/fl^* DSS group were 9.24 ± 1.952 ×10^9^/L, 5.718 ± 1.634 ×10^9^/L, and 1.458 ± 0.518 ×10^9^/L, respectively. In contrast, the *Msi2^ΔRorc^* DSS group had a weaker degree of inflammation, and the numbers of leukocytes, lymphocytes, and monocytes in peripheral blood were 4.08 ± 1.199 × 10^9^/L, 2.34 ± 0.79 × 10^9^/L, and 0.482 ± 0.216 × 10^9^/L, respectively (p < 0.01; p < 0.01; p < 0.01, **Figure 3C**).

These results demonstrate that MSI2 deletion in ILC3s can attenuate histopathological damage and inflammatory responses in DSS-induced mice.

### Changes in the diversity of the intestinal microbiota in the *Msi2^ΔRorc^* mice

Multiple studies of DSS-induced colitis have indicated that the changes in the intestinal microbiota in mice occurred early in inflammation. These changes are characterized by reduced diversity and generally precede clinical or biochemical changes in inflammation. Alterations in the diversity of the intestinal microbiota may lead to the conversion of normally disadvantaged bacteria to dominant bacteria, leading to disturbances in the structure and function of the microbiota (32).

**Figure 4:**
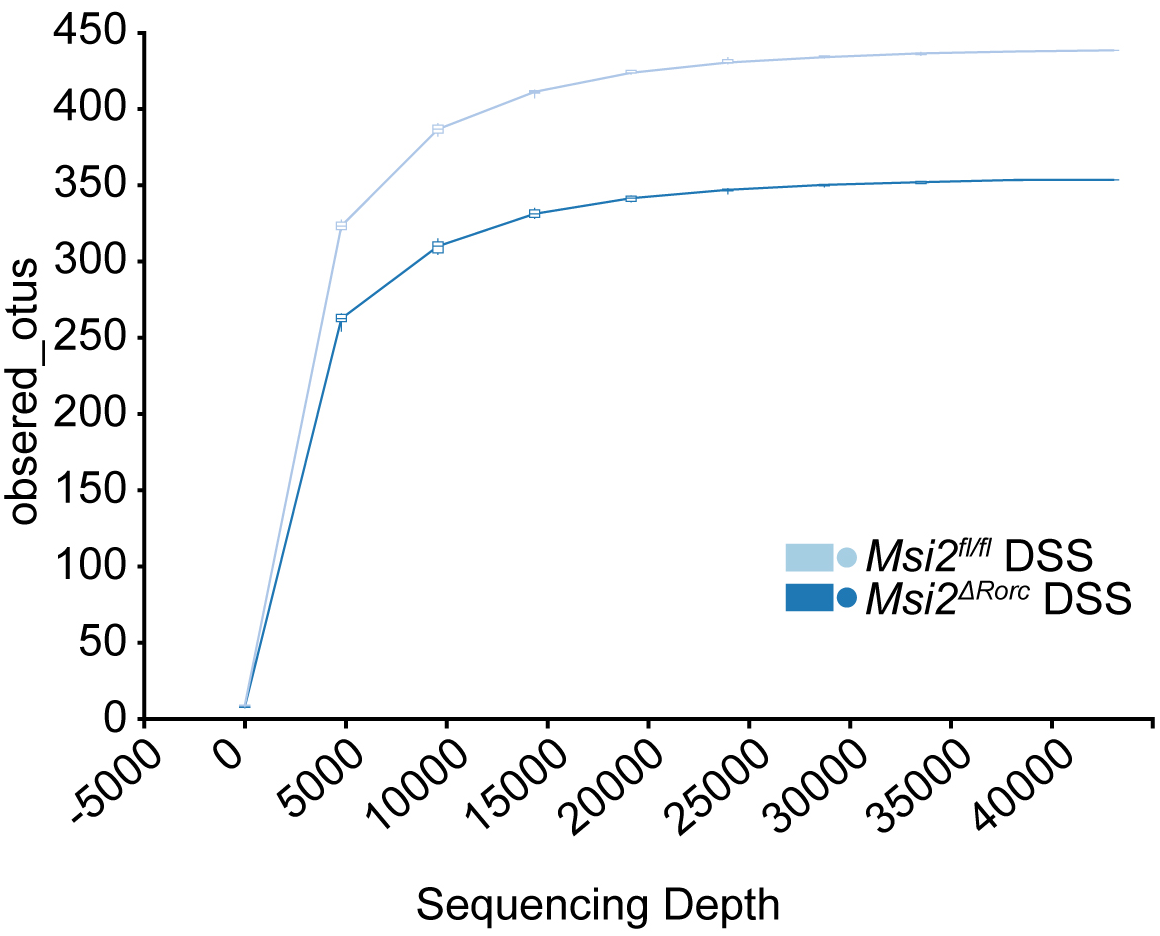
The rarefaction curve for community richness showed that a sufficient sequencing depth was reached (n=8).

To study the effect of MSI2 deletion in ILC3s on the intestinal microbiota diversity of mice, fecal 16S rRNA sequencing technology was used to compare the diversity of the intestinal microbiota in mice before and after DSS administration, including alpha diversity analysis and beta diversity analysis. The rarefaction curve for community richness showed that the sequencing volume covered all intestinal microbiota in the sample and met the data analysis requirements (**Figure 4**). The Shannon and Simpson indices of the intestinal microbiota in the *Msi2^fl/fl^* DSS group were 5.59 ± 0.607 and 0.946 ± 0.016, respectively. In contrast, the alpha diversity of the intestinal microbiota in the *Msi2^ΔRorc^* DSS group was significantly lower, with Shannon and Simpson indices of 4.8 ± 0.572 and 0.89 ± 0.026, respectively. (p < 0.05; p < 0.001).

**Figure 5:**
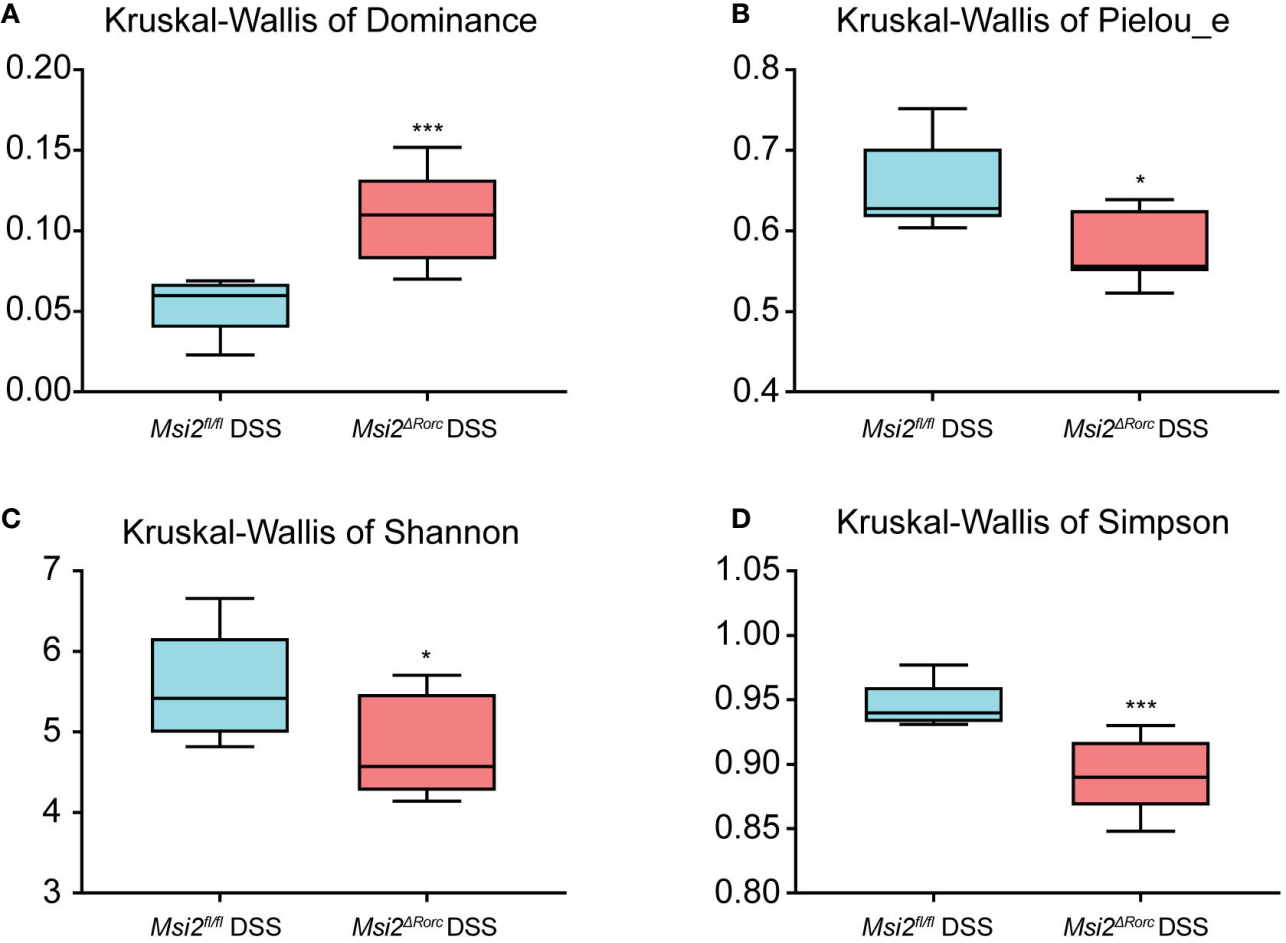
Alpha diversity analysis of the intestinal microbiota between the two groups. Hypothesis tests of the alpha diversity index using the Kruskal−Wallis H test. Dominance, Pielou_e, Shannon, and Simpson **(A–D)** diversity indices between the *Msi2^fl/fl^* DSS group and the *Msi2^ΔRorc^* DSS group confirmed that there were significant disparities in species diversity between these two groups (n=8). *p < 0.05, ***p < 0.001.

**Figure 6:**
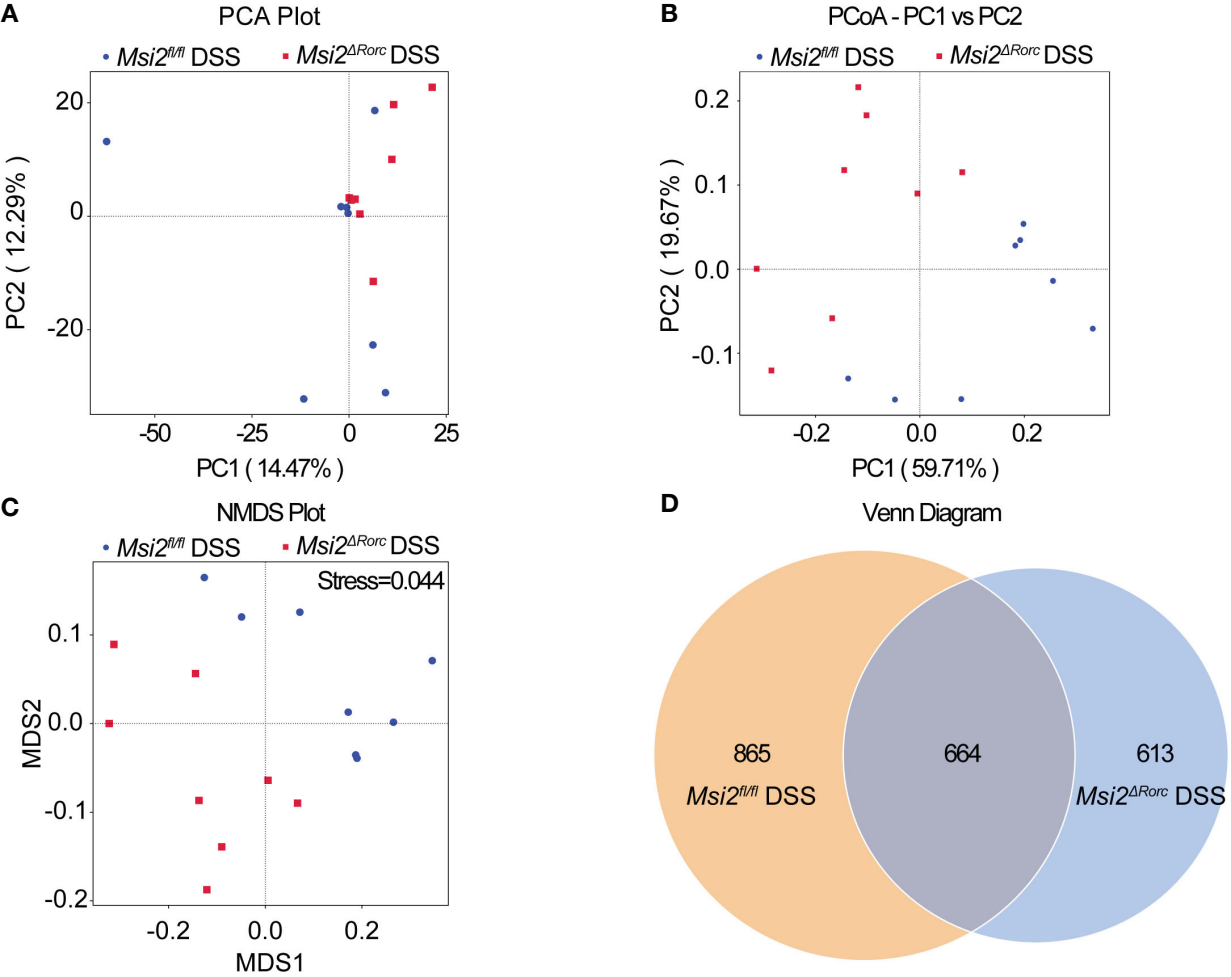
Comparison of the beta diversity index of the intestinal microbiota between the two groups. **(A–C)** PCA indicated a symmetrical distribution of the gut microbial community between the *Msi2^fl/fl^* DSS group and the *Msi2^ΔRorc^* DSS group, which was also confirmed using PCoA and nonmetric NMDS. **(D)** Based on the ASV abundance, a Venn diagram analysis was performed and evaluated. Unique ASVs between the *Msi2^fl/fl^* DSS group (orange) and the *Msi2^ΔRorc^* DSS group (blue) were identified, as well as common ASVs (brown) between the two groups (n=8).

The Pielou_e index and dominance index of the intestinal microbiota of the mice in the experimental group were also analyzed. The results showed that the Pielou_e index of the intestinal microbiota of the *Msi2^fl/fl^* DSS group was 0.654 ± 0.05, and the dominance index was 0.054 ± 0.016. In contrast, the Pielou_e index of the intestinal microbiota in the *Msi2^ΔRorc^* DSS group was significantly decreased to 0.577 ± 0.04, while the dominance index was significantly increased to 0.11 ± 0.026 (p < 0.05, p < 0.001, **Figures 5A–D**). PCA showed that the intestinal microbiota of the *Msi2^ΔRorc^* DSS group and *Msi2^fl/fl^* DSS group were clustered separately, indicating that the structural composition of the intestinal microbiota was significantly different after DSS induction, which was also confirmed by PCoA and NMDS analysis (**Figures 6A–C**).

Each deduplicated intestinal microbiota sequence generated after denoising using statistical methods is called an ASV. ASVs are standard markers artificially set for a taxon (phylum, genus, species, grouping, etc.) to facilitate analysis in phylogenetic or population genetics research. The Venn diagram showed that the number of ASVs in the intestinal microbiota of both the *Msi2^ΔRorc^* DSS group and the *Msi2^fl/fl^* DSS group was 664. In addition, the *Msi2^ΔRorc^* DSS group had 613 specific ASVs in its intestinal microbiota, while there were 865 specific ASVs in the *Msi2^fl/fl^* DSS group (**Figure 6D**).

To further verify whether the structural composition of the intestinal microbiota was significantly different between DSS-treated *Msi2^ΔRorc^* mice and *Msi2^fl/fl^* mice, we performed ANOSIM and Adonis weighted analysis. The results showed that the between-group difference was greater than the within-group difference (p = 0.014925, R = 0.513951), and the grouping factor played a major role in the difference in the diversity of the intestinal microbiota (p = 0.006, R^2^ = 0.357424, **Table 1**).

**Table 1:** ANOSIM and Adonis analysis between groups (n=8).

Comparison	UniFrac	ANOSIM	Adonis
*Msi2^fl/fl^* DSS-*Msi2^ΔRorc^* DSS	Unweighted	p=0.243781 (R=0.037946)	p=0.479 (R^2 =^ 0.064114)
	Weighted	p=0.014925 (R=0.513951)	p=0.006 (R^2 =^ 0.357424)

These results indicate that deletion of MSI2 in ILC3s alters the diversity of the intestinal microbiota in mice after DSS administration.

### Changes in the composition of the intestinal microbiota in the *Msi2^ΔRorc^* mice

Numerous studies have shown that gut dysbiosis plays a crucial role in the pathogenesis of IBD. In DSS-induced colitis, there were significant differences in the composition and abundance of the intestinal microbiota from days 7 to 14, in which the abundance of beneficial bacteria was significantly decreased, while that of harmful bacteria was increased. This trend persisted throughout the experimental period and disappeared by day 21 (33).

**Figure 7:**
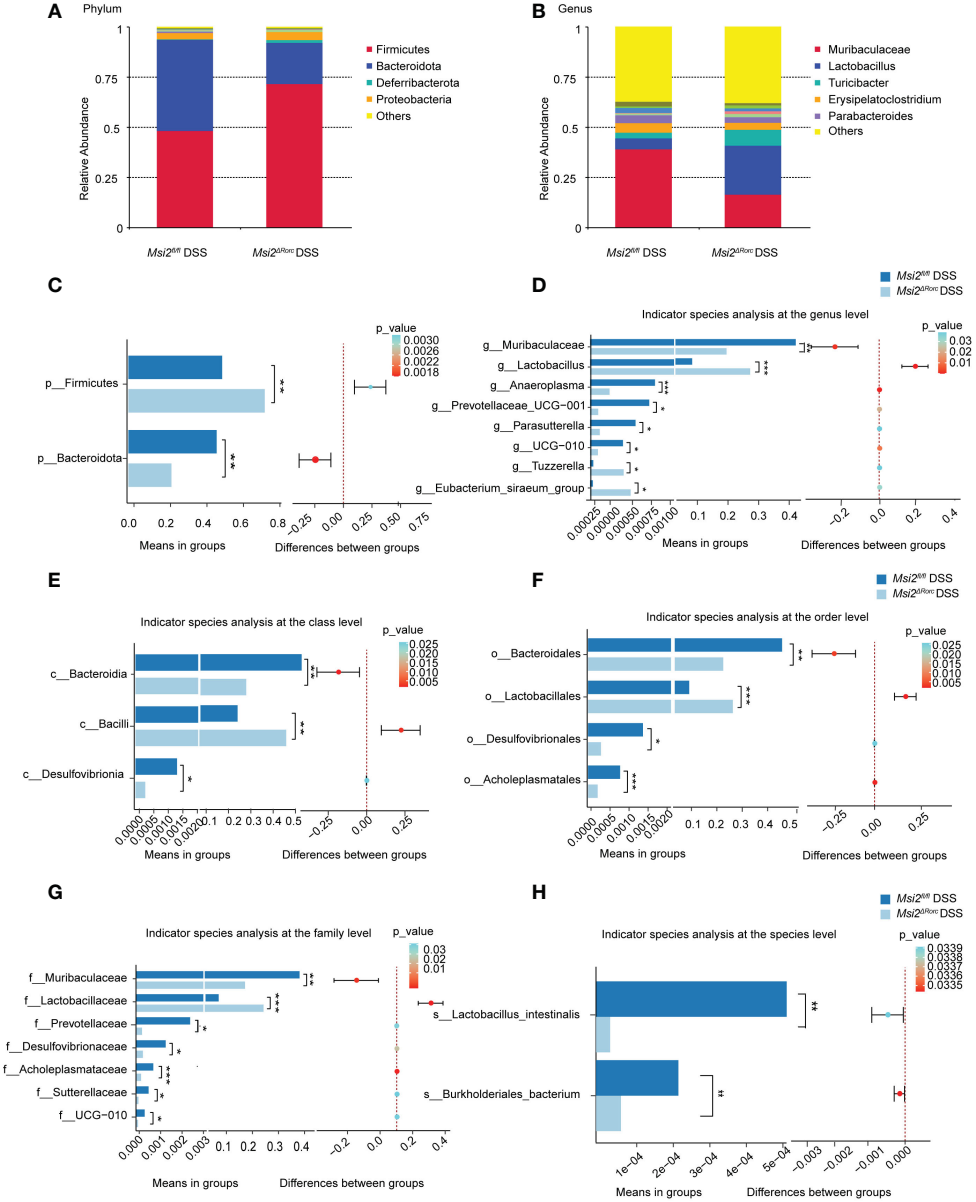
Changes in the intestinal microbiota composition between the two groups. Changes in the relative abundances of the dominant intestinal microbiota were observed at the **(A, C)** phylum and **(B, D)** genus levels between the *Msi2^fl/fl^* DSS group and the *Msi2^ΔRorc^* DSS group. Differentially abundant intestinal microbiota at the **(E–H)** class, order, family, and species levels were observed between the *Msi2^fl/fl^* DSS group and the *Msi2^ΔRorc^* DSS group. ASVs and taxon differences were identified with p values less than 0.05 (n=8). *p < 0.05, **p < 0.01, ***p < 0.001.

To investigate the effect of MSI2 deletion in ILC3s on the intestinal microbiota composition of mice, the intestinal microbiota composition was analyzed by fecal 16S rRNA sequencing. At the phylum level, Firmicutes and Bacteroidota were the most common in the *Msi2^fl/fl^* DSS group and *Msi2^ΔRorc^* DSS group, followed by Deferribacterota, Proteobacteria, Cyanobacteria and Campilobacterota (**Figure 7A**). The abundance of Firmicutes was significantly increased in the *Msi2^ΔRorc^* DSS group, while that of Bacteroidetes was decreased (p < 0.05, **Figure 7C**). At the genus level, Muribaculaceae, Lactobacillus, Turicibacter, Erysipelatoclostridium and Parabacte-roides were the most abundant (**Figure 7B**). The abundances of Lactobacillus, Eubacterium_siraeum_group and Tuzzerella were significantly increased in the *Msi2^ΔRorc^* DSS group, while the genera Muribaculaceae, Anaeroplasma, Prevotellaceae_UCG-001, Parasutterella and UCG-010 decreased in abundance (p < 0.05, **Figure 7D**).

After analyzing the differences in bacterial groups at the phylum level and genus level, we further analyzed whether there were differences in the bacterial composition between the *Msi2^fl/fl^* DSS group and the *Msi2^ΔRorc^* DSS group at the class level. As shown in **Figure 7E**, compared with that in the *Msi2^fl/fl^* DSS group, the abundance of Bacilli was significantly increased in the *Msi2^ΔRorc^* DSS group, while the abundances of Bacterodia and Desulfovibrionia were decreased (p < 0.05, **Figure 7E**). We further analyzed the differences at the order level. The abundance of Lactobacillales was significantly higher in the *Msi2^ΔRorc^* DSS group, while the abundances of Bacteroidales, Desulfovibrionales and Acholeplasmatales were significantly decreased compared with those in the *Msi2^fl/fl^* DSS group (p < 0.05, **Figure 7F**). At the family level, the abundance of Lactobacillaceae in mice in the *Msi2^ΔRorc^* DSS group was significantly increased, while the abundances of Muribaculaceae, Prevotellaceae, Desulfovibrionaceae, Acholeplasmataceae, Sutterellaceae and UCG-010 were decreased compared with those in the *Msi2^fl/fl^* DSS group (p < 0.05, **Figure 7G**). Furthermore, at the species level, the *Msi2^ΔRorc^* DSS group had fewer Lactobacillus intestinalis and Burkholderiales bacterium than the *Msi2^fl/fl^* DSS group (p < 0.05, **Figure 7H**).

**Figure 8:**
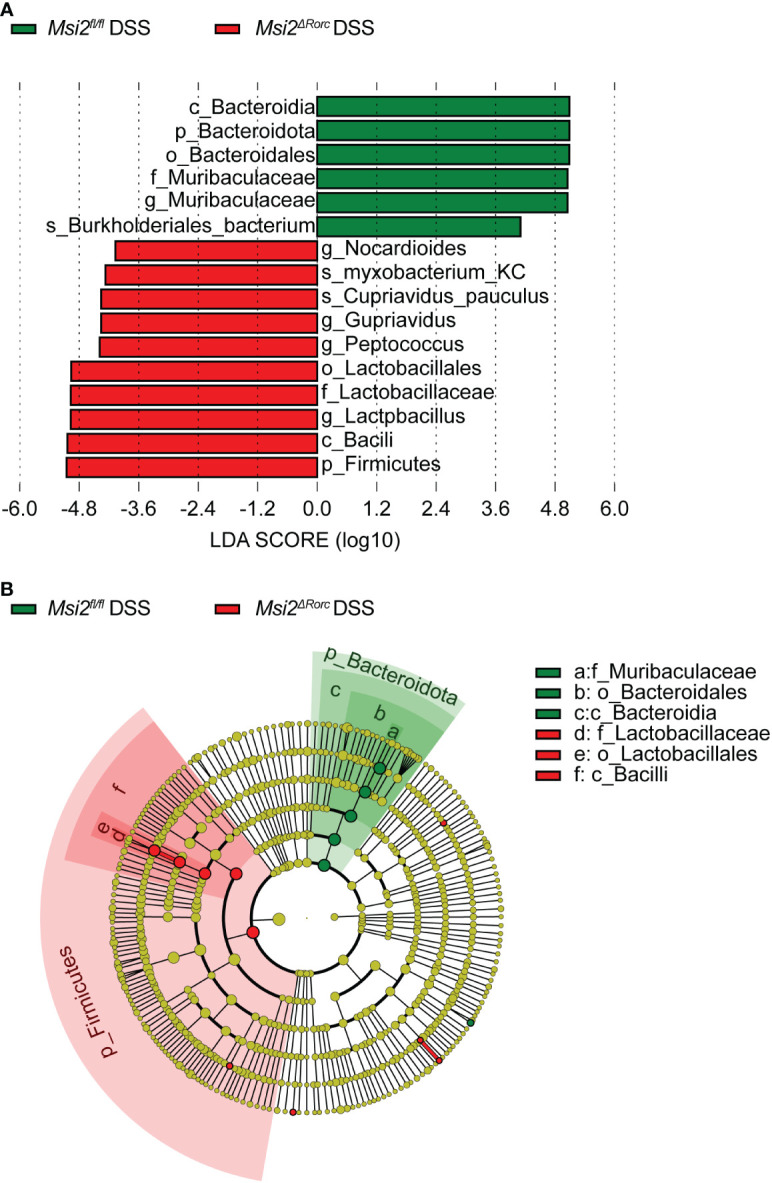
LEfSe analysis indicates the biomarkers of intestinal microbiota between the two groups. **(A)** Taxa with LDA scores greater than 4 according to the comparison of the *Msi2^fl/fl^* DSS group and the *Msi2^ΔRorc^* DSS group. Taxa are represented as p (phylum), c (class), o (order), f (family), g (genus) and s (species). **(B)** Taxonomic cladograms of the *Msi2^fl/fl^* DSS group and the *Msi2^ΔRorc^* DSS group. The green and red dots are proportional to the degree of enrichment of certain taxa between the two comparative groups (n=8).

To further investigate biomarkers of the intestinal microbiota, we applied the LEfSe method to the *Msi2^fl/fl^* DSS group and the *Msi2^ΔRorc^* DSS group. The results showed that Muribaculaceae was the most characteristic bacterial family in the *Msi2^fl/fl^* DSS group, with Burkholderiales_bacterium showing the most significant difference between the two groups. In the classification of bacteria, this family belongs to Bacteroidota, Bacteroidetes, and Bacteroidales. Lactobacillaceae was the most characteristic bacterial family in the *Msi2^ΔRorc^* DSS group, within which Myxobacterium_KC and Cupriavidus_pauculus were the most significantly different between the two groups. In terms of bacterial classification, this family belongs to Firmicutes, Bacilli, and Lactobacillales (**Figures 8A, B**).

### Prediction of changes in the function of the intestinal microbiota in the *Msi2^ΔRorc^* mice

**Figure 9:**
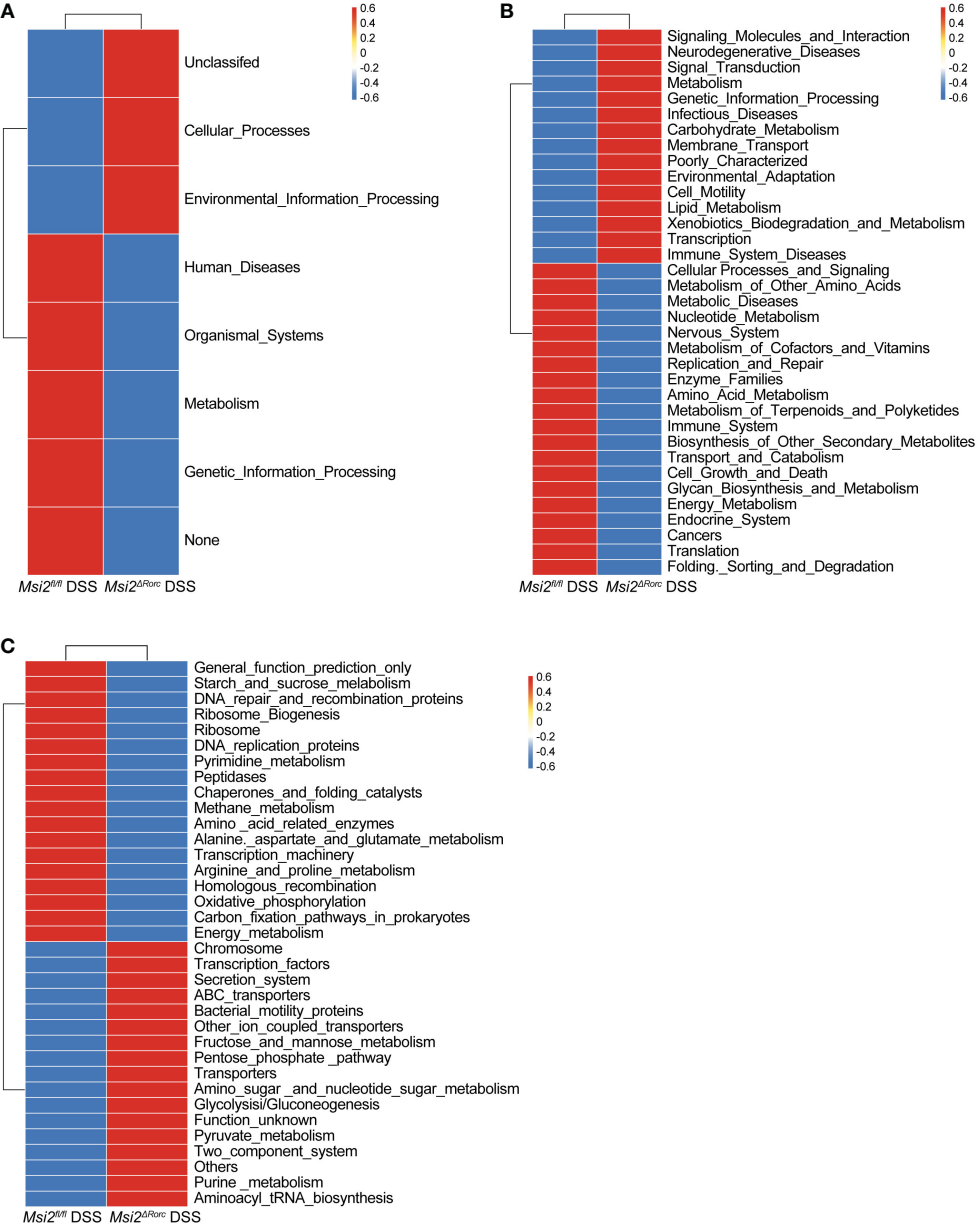
Prediction of the changes in intestinal microbiota function between the two groups. **(A–C)** Heatmap of the changes predicted in intestinal microbiota function between the *Msi2^fl/fl^* DSS group and the *Msi2^ΔRorc^* DSS group in the KEGG pathway analysis at levels 1, 2, and 3 (A-Level 1, B-Level 2, and C-Level 3) (n=8).

With the 16S rRNA gene amplicon sequencing results, PICRUST2 was used for KEGG pathway analysis of the intestinal microbiota (34). Cluster analysis of KEGG pathways at the first level showed that the functions of the intestinal microbiota in the *Msi2^ΔRorc^* DSS group were mainly reflected in cellular processes and environmental information processing. In contrast, the functions of the intestinal microbiota in the *Msi2^fl/fl^* DSS group were mainly reflected in human diseases, biological systems, metabolism, and genetic information processing (**Figure 9A**).

Cluster analysis of KEGG pathways at the second level showed that the gene functions were classified into 35 pathways, among which the functions of the intestinal microbiota in the *Msi2^ΔRorc^* DSS group were mainly reflected in signaling molecules and interactions, neurodegenerative diseases, signal transduction, metabolism, genetic information processing, infectious diseases, carbohydrate metabolism, membrane transport, environmental adaptation, cell motility, lipid metabolism, xenobiotic biodegradation and metabolism and immune system diseases. By comparison, there were some enhanced functions among the intestinal microbiota of the *Msi2^fl/fl^* DSS group, including cellular processes and signaling, metabolism of other amino acids, metabolic diseases, nucleotide metabolism, nervous system, metabolism of cofactors and vitamins, enzyme families, metabolism of terpenoids and polyketides, immune system, biosynthesis of other secondary metabolites, transport and catabolism, cell growth and death, glycan biosynthesis and metabolism, energy metabolism, endocrine system and cancers (**Figure 9B**).

Cluster analysis of KEGG pathways at the third level showed that there were significant differences in the metabolic level of the intestinal microbiota of the experimental group. The functions of the microbiota, such as fructose and mannose metabolism, pentose phosphate pathway, amino sugar and nucleotide sugar metabolism, glycolysis and gluconeogenesis, pyruvate metabolism, purine metabolism and aminoacyl-tRNA biosynthesis, were enhanced in the *Msi2^ΔRorc^* DSS group (**Figure 9C**).

## Discussion

IBD is an inflammatory intestinal disease caused by a variety of etiologies, and the role played by intestinal dysbacteriosis and abnormal immune response cannot be neglected. Our study revealed the influence of the altered diversity and composition of the gut microbiota caused by MSI2 deficiency in ILC3s on IBD. Wang et al. proposed that MSI2 is closely related to the occurrence and progression of colon cancer, and its expression level is significantly increased in colon adenocarcinoma tissues (14). However, the effect of MSI2 on the occurrence and development of IBD has not been reported. This may be because the MSI2 protein is encoded by the *Msi2* gene, and its genetic mutations have been widely reported to be closely related to poor prognosis in cancers. To reveal the possible regulation of MSI2 in IBD, we used DSS-induced colitis to explore the association between MSI2 and IBD. DSS-induced colitis involves intestinal damage mediated by inflammatory cell infiltration and disruption of epithelial structures, consistent with intestinal mucosal surface barrier damage mediated by dysregulated intestinal and acquired immune responses in UC.

In addition, significant amounts of ILC3s and their related cytokines are present in the mucosa of IBD patients (35). ILC3s, as natural lymphocytes corresponding to Th17 cells, express retinoic acid (tretinoin)-related orphan nuclear receptor γt (RORγt), which is distributed in large quantities in the mucous membranes of the ileum and colon and plays an important role in regulating local immunity, inflammation and homeostasis by interacting with the intestinal flora, promoting epithelial cell turnover, stimulating intestinal epithelial cells to produce antimicrobial peptides and regulating adaptive immune homeostasis (36). However, the regulatory effect of MSI2 on innate immune cells has not been reported, and its role in the intestinal immune response and intestinal flora is poorly understood. The effect of MSI2 deletion in ILC3s on the pathogenesis of IBD has not been reported.

In our study, by constructing a DSS-induced colitis model, we found that *Msi2^ΔRorc^* mice were resistant to DSS-induced colitis compared with *Msi2^fl/fl^* mice. First, the intestinal histopathological score of *Msi2^fl/fl^* mice after DSS administration was 4.8 ± 1.17, compared with 2 ± 0.63 in *Msi2^ΔRorc^* mice, and intestinal injury was alleviated. Second, the number of white blood cells in the peripheral blood of *Msi2^fl/fl^* mice after DSS induction was 9.24 ± 1.952×10^9^/L and that of *Msi2^ΔRorc^* mice was 4.08 ± 1.199×10^9^/L. Finally, the colon length of *Msi2^fl/fl^* mice was 5.48 ± 0.35 cm after modeling, while that of *Msi2^ΔRorc^* mice was 7.06 ± 0.72 cm, and the body weight loss of *Msi2^fl/fl^* mice was higher than that of *Msi2^ΔRorc^* mice. These data suggest that MSI2 deficiency in ILC3s attenuates the severity of DSS-induced colitis.

The role of commensal flora in protecting against pathogens has been revealed in the dynamic changes in the intestinal immune system (37). ILC3s, which are widely distributed in the lamina propria of the intestinal mucosa, are important components of the innate immune system. ILC3s stimulate intestinal epithelial cells to secrete antimicrobial peptides by producing the cytokines IL-17 and IL-22, establish a host-intestinal flora symbiotic mechanism, and maintain intestinal homeostasis (38). Another study showed that antigen presentation of ILC3s could directly restrict Th17 cells specific for the gut microbiota and indirectly affect Th1 cells and type 1 immunity involved in the gut microbiota (39). The important role of the gut microbiota and immune regulation in IBD is well established. Certain intestinal bacteria can attach to the intestinal mucosa, invade mucosal epithelial cells, and cause inflammation through TNF-α produced by monocytes and macrophages. The Firmicutes/Bacteroidetes (F/B) ratio is thought to have an important effect on the maintenance of normal intestinal homeostasis. The increase or decrease in the F/B ratio is considered to be an imbalance in flora ecology, with the former manifested as obesity and the latter as IBD (40). Gophna et al. observed a decrease in the Firmicutes abundance in CD patients but not in UC patients or healthy individuals (41). Conversely, the abundance of Bacteroidetes was significantly higher in CD patients than in UC patients and healthy individuals. In addition, Firmicutes were less abundant in active CD and UC patients than in inactive patients. We found that the diversity of the gut microbiota in *Msi2^ΔRorc^* mice was significantly changed after DSS induction. The results of 16S rRNA sequencing showed that compared with *Msi2^fl/fl^* mice, the abundance of Firmicutes was increased and the abundance of Bacteroidetes was decreased in the intestinal microbiota of *Msi2^ΔRorc^* mice. Notably, *Faecalibacterium prausnitzii* in the phylum Firmicutes, which has been proven to have anti-inflammatory effects *in vivo* and *in vitro*, has been observed to decrease in IBD patients (42). This is consistent with our findings, so we believe that the increased Firmicutes/Bacteroidetes ratio in DSS-treated *Msi2^ΔRorc^* mice can effectively reduce the degree of colitis.

In addition, LEfSe analysis showed that Lactobacillaceae was significantly increased in *Msi2^ΔRorc^* mice, and Muribaculaceae was the most significantly different bacterium in *Msi2^fl/fl^* mice. Audrey Y et al. found that the number of protective bacteria including Lactobacillus and Bifidobacterium decreased, while the number of potentially pathogenic bacteria such as Escherichia coli and Clostridium increased in the intestinal microbiota of IBD patients compared with healthy people (43). Lactobacillus can play an important immunomodulatory role by stimulating the synthesis of cytokines, promoting the activation of dendritic cells and natural killer cells, inhibiting the NF-κB pathway, and activating antigen-presenting cells in Pyle’s lymph node, which may be closely related to the improvement in clinical symptoms of IBD (44). As a newly identified bacterium, Muribaculaceae has rarely been reported. Previously known as S24-7 and as “Candidatus Homeothermaceae”, Muribaculaceae has recently been isolated and identified (45). Studies have shown that the family Muribaculaceae belongs to the order Bacteroides and that it can produce propionic acid. Propionate has been reported to inhibit the activation of CD8^+^ T cells, which in turn leads to resistance to immune stimulation (46). The researchers speculated that Muribaculaceae might exert anti-inflammatory effects through propionic acid production, which is inconsistent with our results, and its correlation with inflammation needs to be further studied. In summary, our results indicate that the number of Lactobacillaceae in *Msi2^ΔRorc^* mice increased significantly after DSS administration and that it became the dominant bacteria, which may play an important role in promoting the reduction in inflammation.

Normally, ILC3s play a key role in maintaining crosstalk between the microbiome and the immune environment in intestinal tissues. Goc et al. found that when the activity of normal ILC3s in intestinal tissues is lost, the ability of ILC3s to regulate T cells is significantly disturbed, and the interference of dialogue between ILC3s and T cells will further cause an increase in the level of intestinal inflammation (39). We hypothesize that MSI2 deletion in ILC3s under pathological conditions makes the function of ILC3s more stable, thereby better coordinating the connection between the microbiota and the immune system. However, the effect of MSI2 deletion in ILC3s on the function of ILC3s, as well as on overall innate and adaptive immunity, remains worthy of further study. As a relatively evolutionarily conserved RNA-binding protein, MSI2 synergistically activates or inhibits downstream signaling pathways by inhibiting the translation of target gene mRNAs (47). However, whether MSI2 affects ILC3s through specific downstream signaling pathways or specific molecular targets is still worth further exploration.

## Conclusion

In summary, the deletion of MSI2 in ILC3s can attenuate DSS-induced colonic inflammation in mice by affecting the diversity, composition and function of the intestinal microbiota, and Lactobacillaceae among the Firmicutes could play an important role in this process. This result contributes to our understanding of the interactions among the intestinal microbiota, innate lymphocytes and colitis, which will provide new clues for the prevention and treatment of IBD.

## Data availability statement

The datasets presented in this study can be found in online repositories. The names of the repository/repositories and accession number(s) can be found below: NCBI BioProject, PRJNA836557.

## Ethics statement

The animal study was reviewed and approved by The Institutional Animal Care and Use Committee of Xiamen University.

## Author contributions

NL, ZP, and GZ designed the study. NL, SX, SZ, and QZ conducted the experiments. XM, WA, and YY analyzed the results. BF and ZL collected the clinical samples. NL, SX, SZ, and GZ wrote the manuscript. ZP and HT edited the manuscript and provided critical comments. All authors contributed intellectually to the project through discussion and critically reviewed the manuscript. All authors contributed to the article and approved the submitted version.
