# Supplementary material for: MSI2 deficiency in ILC3s attenuates DSS-induced colitis by affecting the intestinal microbiota
Source: Front Immunol. 2023 Jan 12;13:963379. doi: 10.3389/fimmu.2022.963379 (PMC9877450; doi:10.3389/fimmu.2022.963379)
Supplement: Supplementary file 1 [file DataSheet_1.pdf]

Supplementary Material

1 Supplementary Methods

Methods S1. Immunohistochemical staining (IHC)

Immunohistochemistry was performed on paraformaldehyde-fixed paraffin sections. The following antibodies were used for immunohistochemical staining: CD4 (ab183685, Abcam) (1:100), CD8 (ab217344, Abcam) (1:1000), Foxp3 (12653, CST) (1:200), Ly6G (3344470, Abmart) (1:100), F4/80 (70076S, CST) (1:1000), CD11b (ab1333357, Abcam) (1:1000), and CD11c (97585, CST) (1:200). The sections were dewaxed and dehydrated. In brief, endogenous peroxidase activity was blocked for 10 min using 3.0% hydrogen peroxide, and the sections were blocked for 30 min using 10% goat plasma and then separately incubated with the primary antibodies directed against CD4, CD8, Foxp3, Ly6G, F4/80, CD11b and CD11c at 4 ℃. The staining of the sections was performed using the horseradish peroxidase-streptavidin conjugates of the above primary antibodies (EnVision method). All sections were assessed by an experienced pathologist.

Methods S2. Immunofluorescence staining (IF)

To detect MSI2-expressing RORγt^{+} ILC3s in the colon, we performed immunofluorescence staining. Mouse colon tissues were fixed overnight in 10% formalin followed by routine paraffin embedding and sectioning. In brief, the samples were blocked in Tris-buffered saline (TBS), 1% BSA, 5% donkey serum and 5% goat serum for 1 h at room temperature and then were incubated with anti-CD3 (ab16669, Abcam) (1:100), anti-RORγt (ab207082, Abcam) (1:100) and anti-MSI2 (ab76148, Abcam) (1:100) primary antibodies overnight at 4 ℃. For secondary antibody staining, goat anti-rabbit IgG H&L (HRP) (ab205718, Abcam) (1:2000) was used at room temperature for 1 h. Data were acquired on a Nikon Eclipse Ci-L. Nikon DS-Fi2 was used to collect images.

2 Supplementary Figures

Figure S1. Genotyping and gating strategy for flow cytometry analysis of ILC3s. (A) The DNA levels of *Msi2*$^{fl/fl}$ and *Rorc-Cre* were determined by agarose gel electrophoresis experiments using total DNA extracted from the tails of *Msi2*$^{fl/fl}$ and *Msi2*$^{\Delta Rorc}$ mice. (B) For flow cytometry analyses experiments, single cells were first gated on FSC-A vs. SSC-A, and doublets were excluded by gating on FSC-A vs. FSC-H. Live immune cells were further gated on minimal fluorescence with a fixable viability dye and CD45 expression. Flow cytometry gating strategy to determine the percentage of CD127^{+}RORγt^{+}ILC3 cells in the CD45^{+}LIN^{-} lymphocyte population of the colon in *Msi2*$^{fl/fl}$ and *Msi2*$^{\Delta Rorc}$ mice. (C) Colonic RORγt^{+}ILC3s from *Msi2*$^{fl/fl}$ Control and *Msi2*$^{\Delta Rorc}$ Control mice

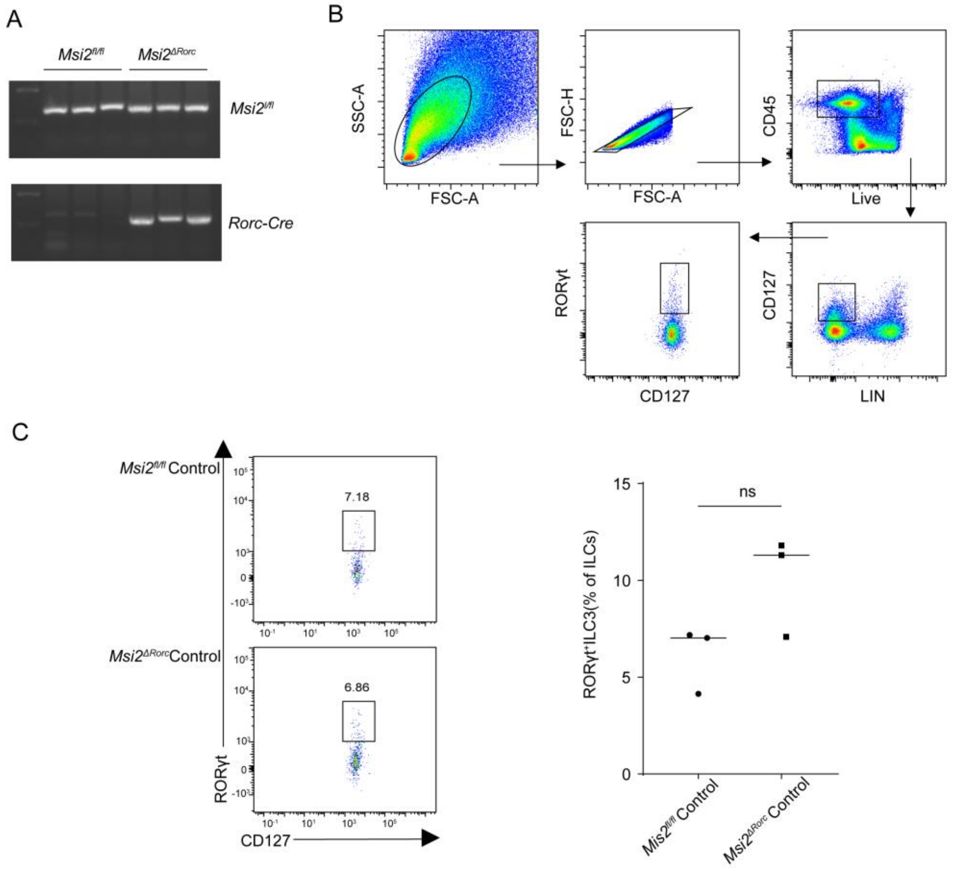

Figure S2. Rarefaction curve for community richness showed that sufficient sequencing depth was reached (n=8).

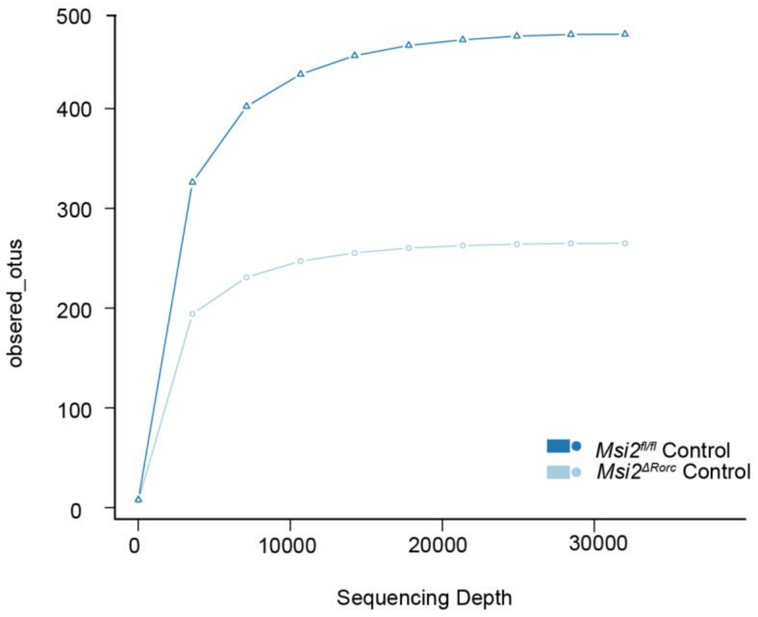

Figure S3. Alpha diversity analysis of intestinal microbiota between two groups. Hypothesis tests of the alpha diversity index through Kruskal-Wallis H test. Dominance, Pielou_e, Shannon, Simpson (A-D) diversity indexes between the *Msi2*$^{fl/fl}$ Control group and the *Msi2*$^{\Delta Rorc}$ Control group confirmed there were no significant disparities in species diversity between these two groups (n=8).

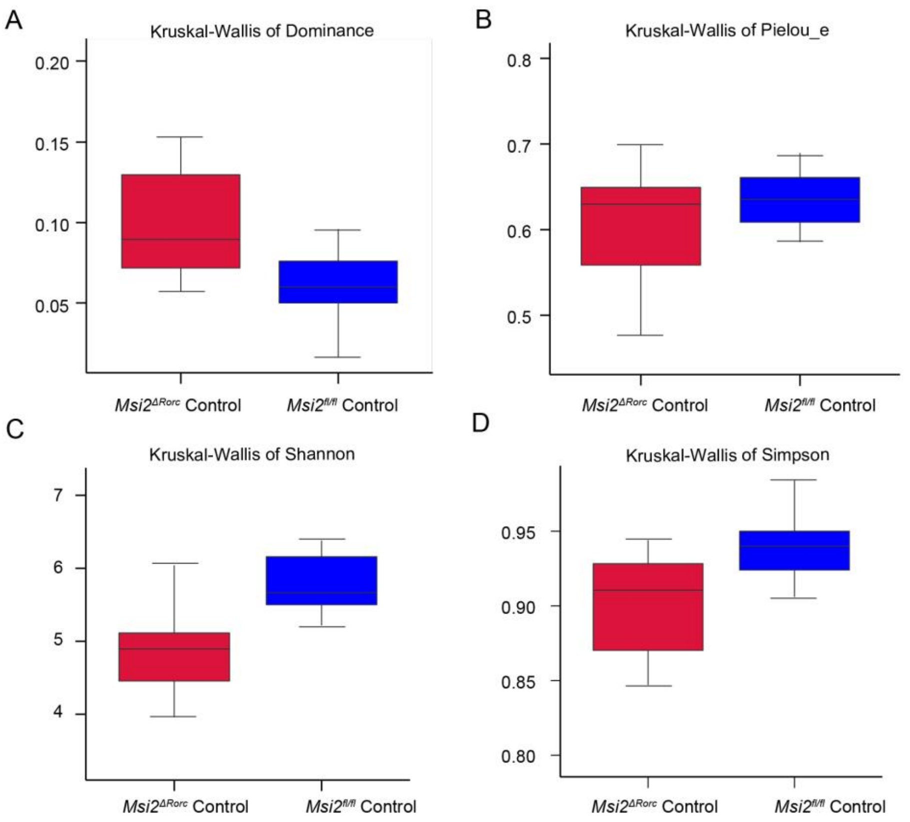

Figure S4. Comparison of beta diversity index of intestinal microbiota between two groups. (A-C) PCA indicated a symmetrical distribution of the intestinal microbial community between the *Msi2*$^{fl/fl}$ Control group and the *Msi2*$^{\Delta Rorc}$ Control group, which was also confirmed using PCoA and nonmetric NMDS. (D) Based on the ASV abundance, a Venn diagram analysis was performed and evaluated. Unique ASVs between the *Msi2*$^{fl/fl}$ Control group (brown) and *Msi2*$^{\Delta Rorc}$ Control group (blue) were identified, as well as common ASVs (dark blue) between the two groups (n=8).

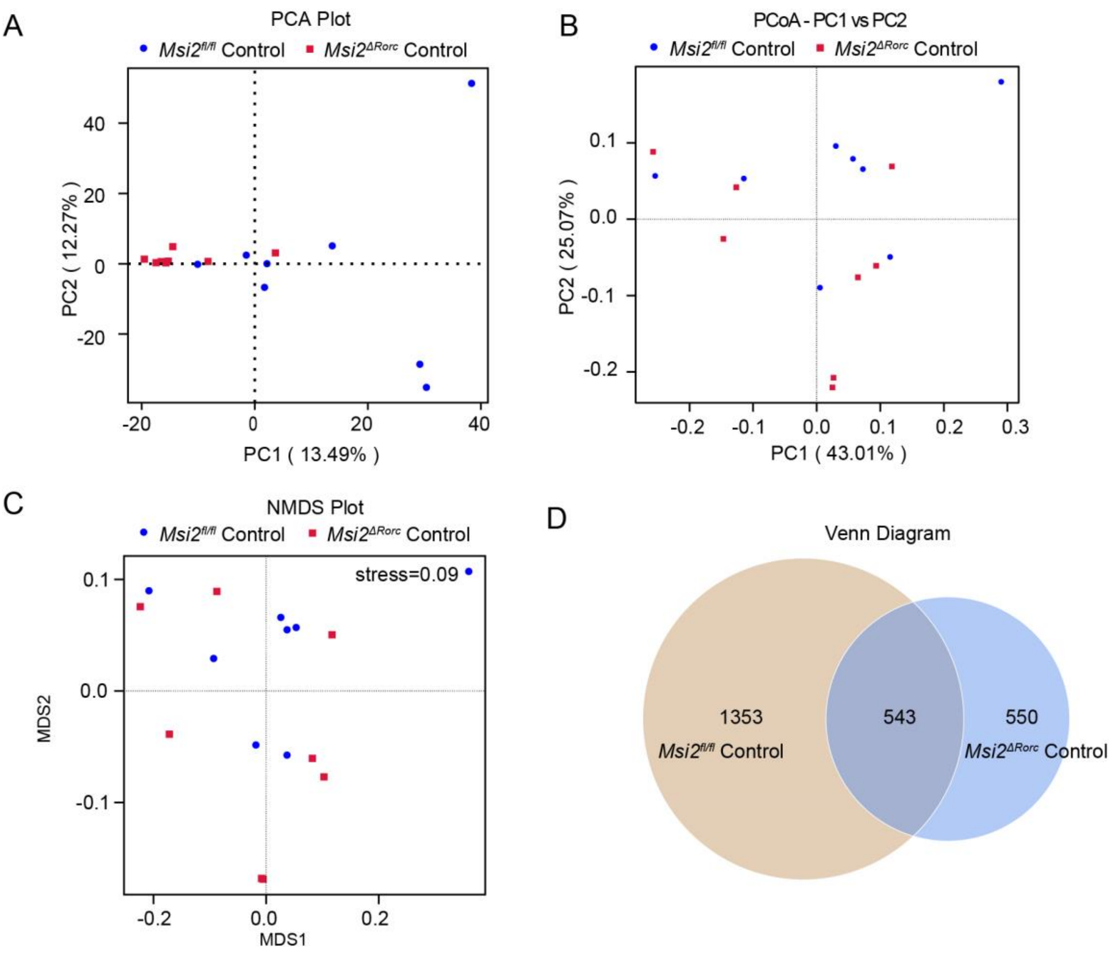

Figure S5. Changes in the intestinal microbiota composition between two groups. There are no dominant intestinal microbiota changes were seen at the (A, C) phylum and (B, D) genus levels between the *Msi2*$^{fl/fl}$ Control group and the *Msi2*$^{\Delta Rorc}$ Control group. Almost no difference among the intestinal microbiota at (E-H) class, order, family, and species-level changes were seen between the *Msi2*$^{fl/fl}$ Control group and the *Msi2*$^{\Delta Rorc}$ Control group. ASVs and taxa differences are identified with no significant disparities (n=8).

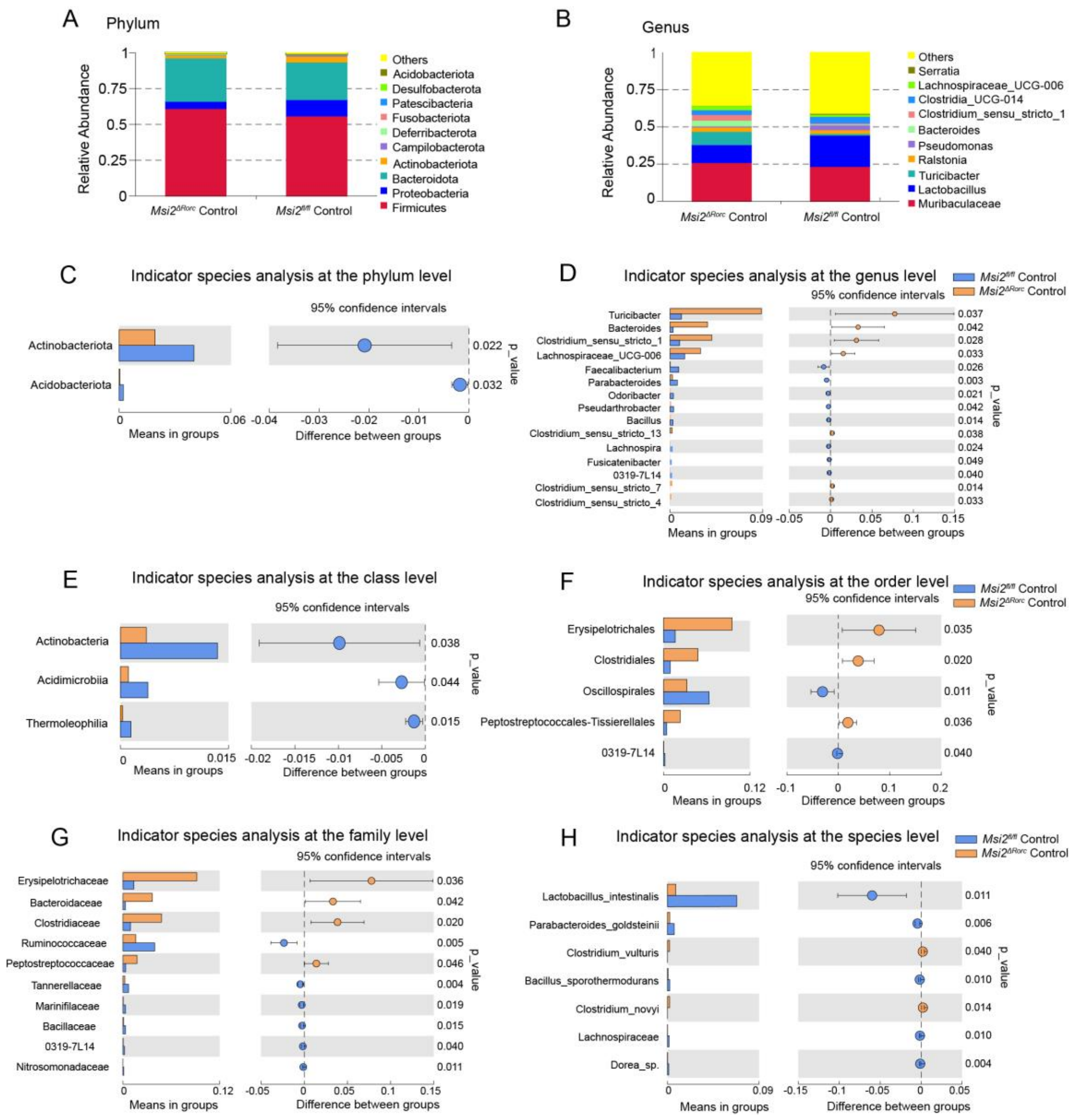

Figure S6. LEfSe analysis indicates the biomarkers of intestinal microbiota between two groups. (A) Taxa with LDA scores greater than 4 according the comparison of the *Msi2*$^{fl/fl}$ Control group and the *Msi2*$^{\Delta Rorc}$ Control group. Taxa are represented as p (phylum), c (class), o (order), f (family), g (genus) and s (species). (B) The taxonomic cladograms of the *Msi2*$^{fl/fl}$ Control group and the *Msi2*$^{\Delta Rorc}$ Control group, the green and red dots are proportional to the degree of enrichment of certain taxa between the two comparative groups (n=8).

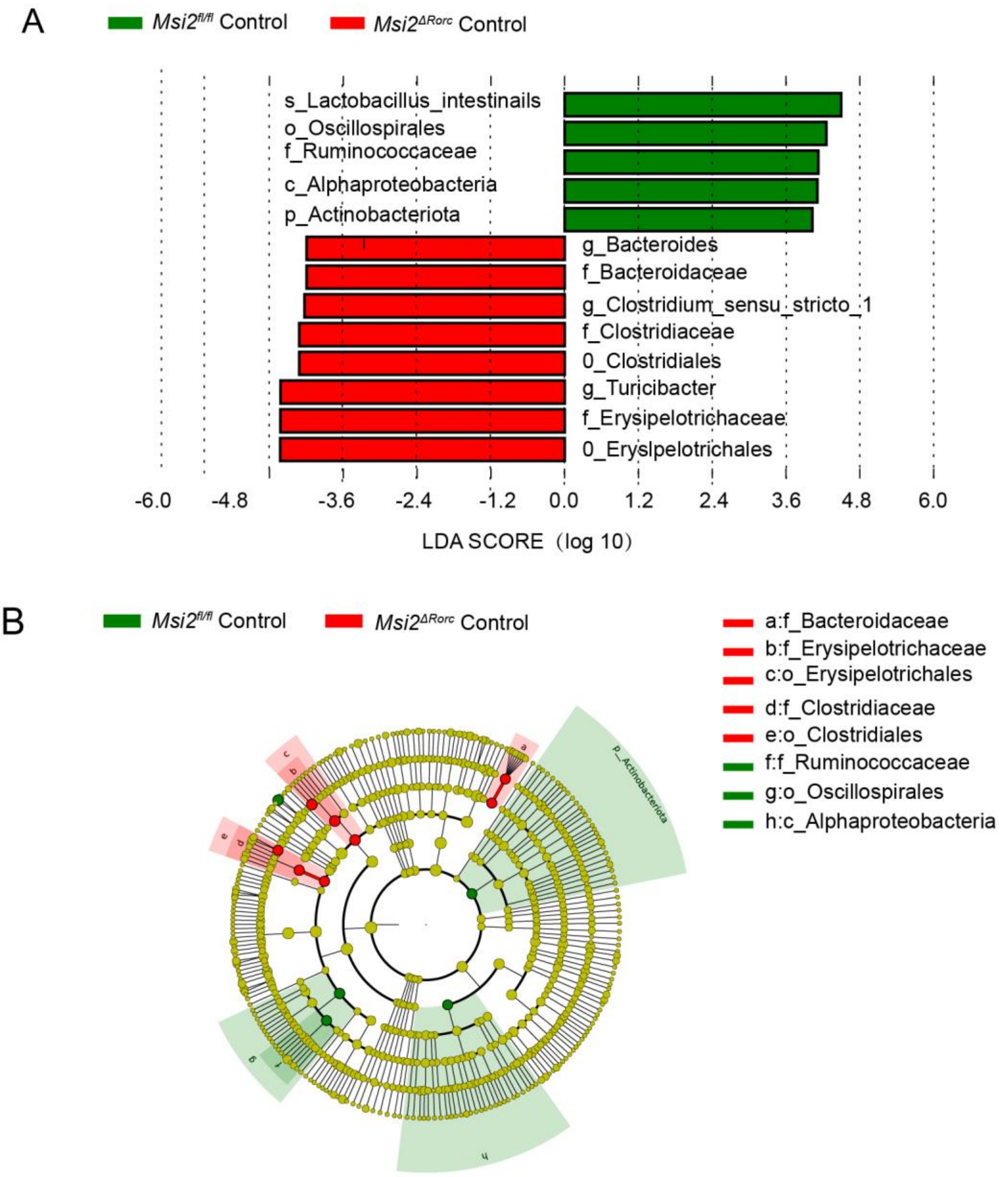

Figure S7. Predict the changes in the intestinal microbiota function between two groups. (A-C) The heatmap of the changes predicted in the intestinal microbiota function between the *Msi2*$^{fl/fl}$ Control group and the *Msi2*$^{\Delta Rorc}$ Control group in KEGG pathway at levels 1/2/3(A-Level 1, B-Level 2, C-Level 3) (n=8).

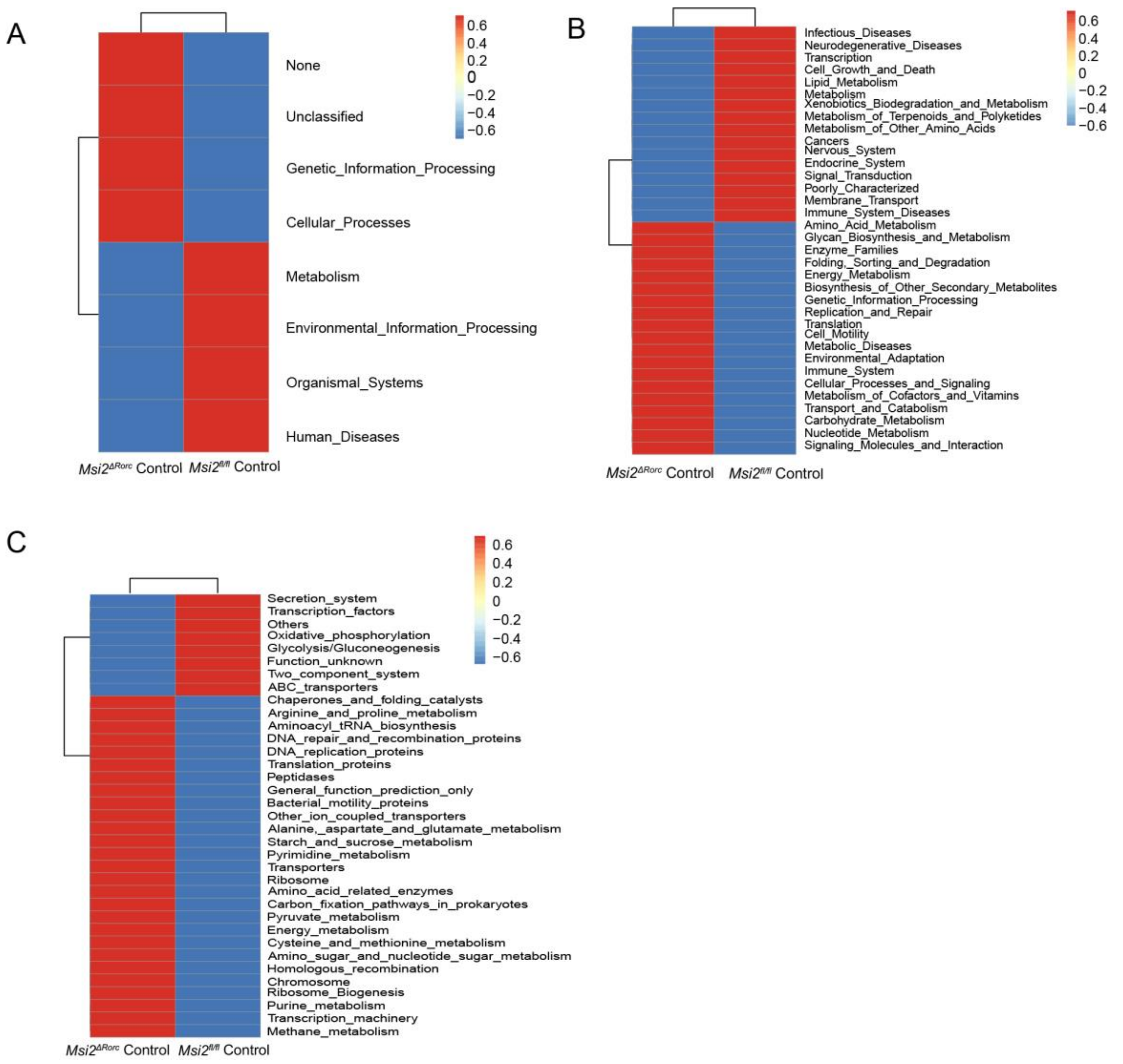

3 Supplementary Table

Table S1. ANOSIM **and Adonis analysis between groups (n=8).**

**Comparison**	**UniFrac**	**ANOSIM**	**Adonis**
$Msi2^{fl/fl}$ Control - $Msi2^{\Delta Rorc}$ Control	Unweighted	p=0.02 (R=0.371094)	p=0.01 (R^2=0.17025)
	Weighted	p=0.29 (R=0.03125)	p=0.18 (R^2=0.10082)
